# Comparative performance of between-population vaccine allocation strategies with applications to SARS-CoV-2

**DOI:** 10.1101/2021.06.18.21259137

**Published:** 2022-01-25

**Authors:** Keya Joshi, Eva Rumpler, Lee Kennedy-Shaffer, Rafia Bosan, Marc Lipsitch

**Affiliations:** a Center for Communicable Disease Dynamics, Department of Epidemiology, Harvard TH Chan School of Public Health, 02115 Boston, Massachusetts; b Department of Mathematics & Statistics, Vassar College, 12604 Poughkeepsie, New York

**Keywords:** vaccine allocation, vaccine distribution, emerging pathogen, COVID-19

## Abstract

Vaccine allocation decisions during the ongoing COVID-19 pandemic have proven to be challenging due to competing ethical, practical, and political considerations. Complicating decision making, policy makers need to consider vaccine allocation strategies that balance needs both within and between populations. Due to limited vaccine stockpiles, vaccine doses should be allocated in locations where their impact will be maximized. Using a susceptible-exposed-infectious-recovered (SEIR) model we examine optimal vaccine allocation decisions across two populations considering the impact of population size, underlying immunity, continuous vaccine roll-out, heterogeneous population risk structure, and differences in disease transmissibility. We find that in the context of an emerging pathogen, such as SARS-CoV-2 where many epidemiologic characteristics might not be known, equal vaccine allocation between populations performs optimally in most scenarios. In the specific case considering heterogeneous population risk structure, first targeting individuals at higher risk of transmission or death due to infection leads to equal resource allocation across populations.

## 3 Introduction

Since its emergence, SARS-CoV-2 has caused considerable suffering and disruption of societies, resulting in over 281 million infections and 5.4 million deaths worldwide as of December 2021.^1^ Vaccines are currently the most effective public health intervention available against SARS-CoV-2, with nine vaccines currently approved for full use and an additional sixteen authorized for early or limited use globally.^2,3^

Even with the approval of multiple vaccines, distributed across different regions globally, roll-out has been slow. While some countries are falling far behind their anticipated timelines, vaccination rates are highly unequal across countries.^4^ Indeed, as some countries have already vaccinated over 90% of their population, and are on track to vaccinate all willing adults by the end of 2021, others still do not have access to vaccines.^4^ Even as COVAX– a Coalition for Epidemic Preparedness Innovations (CEPI), Gavi the Vaccine Alliance (GAVI) and World Health Organization (WHO) collaboration – aimed to deliver approximately 1.4 billion doses to 92 low- and middle-income economies by the end of 2021,^5^ it could take up to 2023^6,7^ for all countries to have access to a sufficient number of doses. Overall, vaccination allocation decisions are currently being made under the constraint of a limited vaccine stockpile and multiple factors need to be considered to maximize the effect of each dose across populations.

As previous work^8–10^ has shown, targeting specific subgroups within a given population, including older individuals, results in decreased COVID-19 morbidity and mortality. Further, previous theoretical work^11^ has shown that unequal vaccine allocations might be favorable in emerging infectious disease settings, but are less optimal when incorporating realistic assumptions about population heterogeneity and contact structure. This leaves a potentially conflicting message for policy makers when considering optimal allocation decisions. We build upon this work, by not only considering the optimal decision, but also how the decision compares to all possible allocations across two populations. Illustrating these tradeoffs with a simplified model of the spread and progression of SARS-CoV-2 infection, our results show that the efficiency gains for unequal allocations that are found in models with highly simplified epidemics are typically small; moreover, they vanish and can even reverse under settings more relevant to the current pandemic. Similar to previous findings, we show in more realistic scenarios, incorporating population heterogeneity and interaction between populations, equal distributions are not only optimal, but vastly outperform unequal distributions.

## 4 Methods

We use a deterministic, two-population, susceptible-exposed-infectious-recovered (SEIR) compartmental model. We assume people are initially susceptible (S). Susceptible individuals move to the exposed state (E) after an effective contact with an infectious individual. After a latent period, exposed individuals become infectious (I). After the infectious period has elapsed, infectious individuals move to a recovered state (R). We do not account for waning immunity and assume once individuals have recovered, they stay immune to infection for the duration of our simulation, here modeled as three years. We start by assuming that there is no interaction between the two populations, so all disease transitions happen in parallel between the two populations.

We extend this SEIR model to allow for underlying immunity and vaccination (Figure S8). At the start of the epidemic, in each population, individuals can be in the susceptible (S), infectious (I), or recovered (R) compartments. When there is underlying immunity, a set proportion of individuals are placed in R. Individuals in R, whether through underlying immunity or infection through the course of the simulation, can never be re-infected. When vaccine doses are distributed to the population, vaccinated individuals are placed in R if the vaccination is successful. We assume that the vaccine is all-or-nothing with 95% efficacy, meaning 95% of those who are vaccinated are placed in R and the remainder stay in S. When there is underlying immunity and vaccination, immune individuals may be vaccinated; vaccination has no effect on them, and they remain in R. Finally, we initialize each simulation by placing 0.1% of individuals in I and allow the epidemic to run, unmitigated except by vaccination, through each population. Full model parameters and equations are shown in SI Appendix A.2. Where possible, parameters represent estimates from the COVID-19 pandemic; for example, 95% vaccine efficacy is similar to that estimated for the Moderna mRNA vaccine Spikevax.^12^

To recreate the results of Keeling and Shattock^11^ we model two homogeneous populations with identical characteristics apart from population size. In this scenario we assume population 2 is double the size of population 1 (Figure 2). For later scenarios, which consider the impact of heterogeneity within populations, we simulate two populations that are identical in size, but vary in their population characteristics (e.g. fraction high risk) (Figure 5, S3–S7).

Next, we extend the model by allowing for heterogeneous risk groups. Within each population, we first model efficient transmitters of infection, such as young adults for the SARS-CoV-2 virus.^13^ In this scenario, we assume high-transmitters are four times more likely to transmit compared to low transmitters.^14^ We fix the within-population structure to allow the global *R*_0_ to equal 2, 4, 8 or 16. The full derivation is shown in SI Appendix A.2.5.

In addition, we also model individuals at elevated risk of death from infection; this can represent, for example, elderly individuals or other individuals with co-morbidities known to exacerbate COVID-19 disease.^15,16^ For simplicity of the model, we assume these individuals are five times more likely to die than other individuals infected with SARS-CoV-2^17^. Note that death rates are assumed to be constant throughout the epidemic, which may not be realistic as health care resources are strained by large caseloads or case management improves over time.

Similar to the homogeneous two population scenario described above, we initialize the model by placing individuals from each population in the susceptible or recovered state based on the pre-existing immunity level and the vaccine allocation scenario. The total number of vaccine doses are split amongst the two populations based on the scenario. Within each population, high-risk individuals are vaccinated first, with leftover doses then allocated to the low-risk population, as described in SI Appendix A.2.6.

Finally, we model the scenario where vaccines are unavailable at the start of the epidemic but are slowly rolled out over the course of the epidemic. For this simulation we vary both the timing of roll-out, and the fraction of the population vaccinated each day. We allow vaccine roll-out to start 1, 10, 30, 50, or 100 days after the epidemic has begun and vary the proportion of the population vaccinated from 1% to 3% per day. For these simulations, the vaccine is allocated within and across populations identically to the scenarios described above for the homogeneous and heterogeneous scenarios.

For each simulation we calculate the cumulative number of infections and deaths from the deterministic SEIR model at the end of the epidemic. Across each scenario we define the optimal allocation strategy as the one that minimizes the total epidemic size (cumulative number of infections) across both populations. Within the high morbidity scenario, we define the optimal allocation strategy as the one that minimizes the total number of deaths across both populations. This is equivalent to maximizing the total number of people across both populations that escape infection (or death).^18^

We conduct sensitivity analyses to assess the robustness of our results. First, we model a leaky vaccine scenario where we assume the vaccine reduces susceptibility to infection by 95%. As a result, all vaccinated individuals (except those previously immune through natural infection) can become infected with the virus, although the probability of infection for each contact with an infected individual is lower than for an unvaccinated individual. We further extend the model by relaxing the assumption that the two populations do not interact. We allow a fraction *i* of infected individuals in both populations to contribute to the force of infection in the other population instead of their own population. An *i* value of 0 corresponds to no interaction, and an *i* value of 0.5 corresponds to complete interaction between the two populations (i.e. is equivalent to one large population). Finally, for each of the scenarios above, we additionally consider the impact of varying *R*_0_ between 2 and 16, allowing for improved understanding across a variety of pathogens or across SARS-CoV-2 variants.^19^ Full model equations are shown in SI Appendix A.2.

All analyses were conducted in R version 4.0.3.

## 5 Results

### 5.1 Literature review

We reviewed the literature on optimal vaccine allocation across populations that was published prior to the emergence of SARS-CoV-2 (see SI Table A.3.1). Multiple papers^11,20–23^ have shown that allocation proportional to population size is rarely optimal. Further, previous studies have highlighted that the timing of vaccine allocation,^24–26^ heterogeneity in population composition, as well as the stochasticity in infection dynamics affect the optimal distribution.^27,28^ Duijzer et al.^18^ provide important contributions by showing that the optimal vaccination threshold is often not the herd immunity threshold as further detailed in SI Appendix A.3.3.

Additionally, we summarize some of the recent literature modeling optimal SARS-CoV-2 vaccine allocation in one population in SI Table A.3.2.

### 5.2 Optimal allocation in two populations of equal size

**Figure 1:**
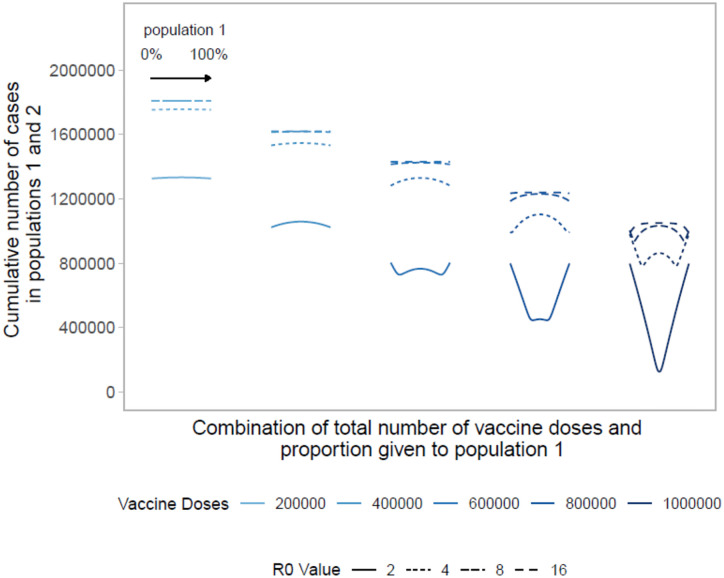
Performance of different allocation strategies of a limited SARS-CoV-2 vaccine stockpile across two homogeneous population of equal size with no underlying immunity and prophylactic vaccination. Both populations have one million individuals. Each color represents a different number of total vaccine doses. Each line represents a different basic reproductive number. Each curve shows the cumulative number of COVID-19 cases across both population 1 and 2 for different proportions of doses given each population. Across each curve, the proportion of doses to population 1 goes from 0 to 100%.

We build upon the existing literature by first examining allocation decisions in the simple scenario of two identical, non-interacting populations with no underlying SARS-CoV-2 immunity (see Figure 1). In the simplest case, with a small number of vaccine doses available, pro-rata allocation performs comparably to highly unequal allocation strategies. As the number of vaccine doses increases, highly unequal strategies gain advantage over pro-rata allocation. This occurs because one population can be vaccinated close to, but lower than, its herd immunity threshold, maximizing the indirect effect of the vaccine doses.^18^ When sufficient vaccines are available for both populations to reach that threshold, more unequal strategies use the doses less efficiently, as indicated by the increasing arms of the “W” shapes in Figure 1. Allocating doses to the population that has reached its threshold provides limited benefit in that population and withholds doses from the other. When there are nearly enough doses to reach the thresholds in both populations, the optimal strategy becomes equal allocation between the two populations.

As the basic reproductive number increases, we again see that unequal allocations preform optimally, as the number of available doses is less than the number needed to reach the critical herd immunity threshold in both populations. In these scenarios, vaccinating one or the other population until it can reach the critical herd immunity threshold results in the lowest cumulative cases across both populations. At very high basic reproductive numbers (i.e., *R*_0_ = 16), pro-rata allocation performs comparably to highly unequal allocation strategies.

### 5.3 Optimal allocation in two populations of unequal size

Extending the simple case of non-interacting populations of equal size, previous studies have shown how optimal allocation across populations of different sizes is not linear, but varies with the number of doses available in a characteristic, and often counter-intuitive, “switching” pattern.^11,18,21^

**Figure 2:**
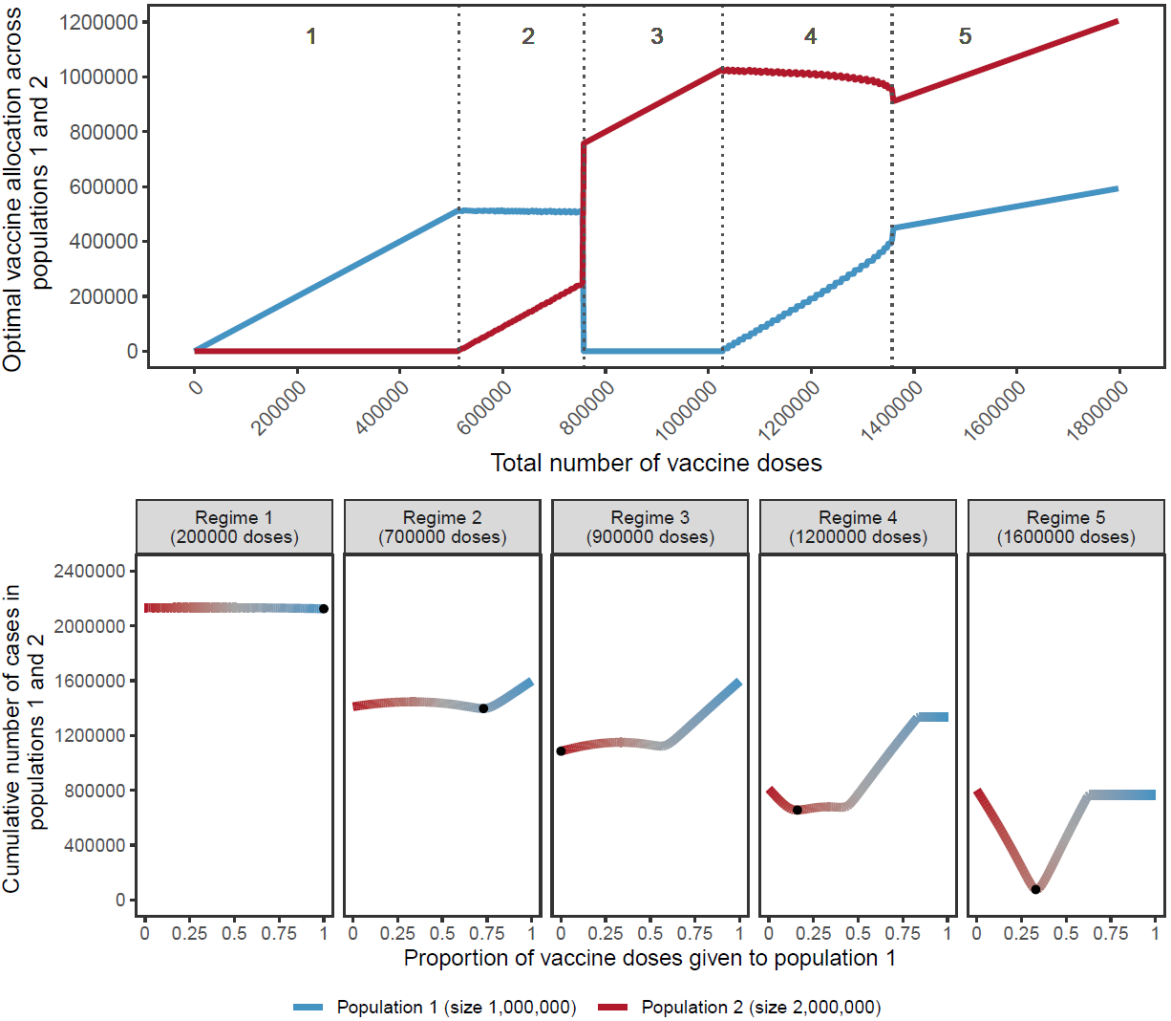
Top: Optimal allocation strategies of a limited SARS-CoV-2 vaccine stockpile across two homogeneous populations of unequal size with no underlying immunity, prophylactic vaccination and an R_0_ of 2. Populations 1 (blue) and 2 (red) have one and two million individuals, respectively. Dashed vertical lines were added to highlight regimes (1 to 5) showing different vaccine allocation patterns. Bottom: Performance of allocation strategies for five different numbers of vaccine doses, representative of the regimes shown in the top half of the Figure. Color coding corresponds to vaccine allocation ranging from giving all doses to population 2 (red) to giving all doses to population 1 (blue). The optimal allocation, the minimal value on each plot, is highlighted by a black point.

As shown in Figure 2 (top), when the number of doses available is very limited, optimal allocation concentrates all vaccine doses to the smallest population, not assigning any to the largest population (regime 1). As the number of doses allocated to the smaller population reaches its threshold, additional doses are gradually allocated to the larger population (regime 2). Strikingly, for 757,500 doses available, a drastic switch happens, and all doses are allocated to the larger population and none to the smaller one (regime 3). Then, as the largest population itself reaches its threshold, supplementary doses are assigned to the smaller population (regime 4). When the number of vaccines available allows both populations to attain their respective thresholds, vaccines are allocated proportionally to the population sizes (regime 5). Note that here we assume the total number of doses is fixed at the time of allocation and no additional doses become available over time. We relax this assumption in later scenarios considering continuous rollout.

For most values of vaccine available, the optimal allocation is highly unequal, as previously shown.^11,18^ This counterintuitive result is caused by the non-linearity of the indirect effect from each additional vaccine dose. Additional doses are allocated to the population where they have the largest benefit. For example, in regime 1 of Figure 2, additional doses bring a larger benefit in the smaller population then they would in the larger population.

Importantly, while prior literature^11^ demonstrates that unequal allocations can be optimal, these results show that the benefit of such unequal, optimal allocations over more nearly equal ones is often small. As shown in Figure 2 (bottom), for low numbers of vaccine doses (regimes 1 and 2), although concentrating all doses to the smallest population is optimal, other strategies do not perform much worse. Each regime is characterized by a different allocation profile that gives rise to a different optimum, indicated by black points. In regime 4, the characteristic W shape appears where a fully unequal allocation is sub-optimal, regardless of which population is vaccinated.

### 5.4 Impact of underlying immunity

As vaccines become available to different locations at different points in their local epidemic, populations will have varying degrees of underlying immunity to the virus due to prior infections. Serological surveys estimate that around a fifth of the population had already been infected in areas hardest hit during the spring of 2020 (23% in NYC^29^, 18% in London^30^ and 11% in Madrid and Paris^31,32^). Select high-risk groups, including health care workers and nursing home residents, have been shown to have an even higher prevalence of SARS-CoV-2 antibodies.^33^ To account for underlying immunity, we further simulate optimal allocation decisions with varying levels of underlying immunity in each population to mirror the fact that allocation decisions are made during an ongoing pandemic.

**Figure 3:**
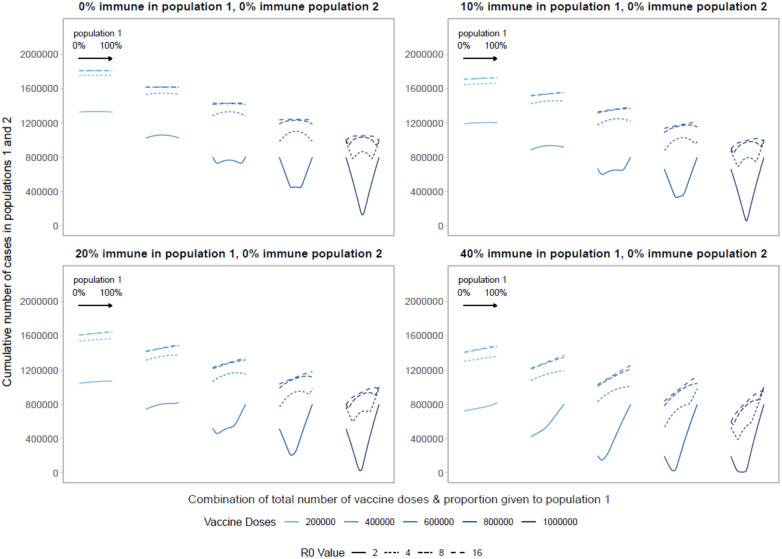
Performance of different allocation strategies of a limited SARS-CoV-2 vaccine stockpile across two homogeneous populations of equal size (one million individuals) with different underlying immunity, and prophylactic vaccination. We fix population 2 to have no underlying SARS-CoV-2 immunity and vary underlying immunity in population 1 from 0 to 40%. Each color represents a different number of total vaccine doses. Each line represents a different basic reproductive number. The panel on the top left is equivalent to Figure 1.

Comparing two populations with varying amounts of underlying immunity, the optimal strategy favors prioritizing the population that is closer to their herd immunity threshold (Figure 3). Figure 3 shows optimal allocation decisions across two homogeneous populations of equal size with no immunity (top left, repeating Figure 1) or increasing degrees of immunity in population 1. With increasing immunity in population 1, the characteristic V- or W-shape becomes more lopsided as fewer doses are required in population 1 to reach the threshold at which doses should be split between populations. Extremely unequal allocation strategies either waste doses or fail to minimize the cumulative number of infections in both populations if given completely to population 1 or 2, respectively. In addition, allocating vaccines to population 1 beyond the amount needed to reach its threshold results in the highest cumulative number of COVID-19 cases because it confers little additional benefit in population 1, and deprives population 2 of vaccines needed to mitigate cases. As before, once the number of doses is large enough to approach or reach the threshold in both populations, optimal strategies move closer to pro-rata allocations.

As we vary the basic reproductive number, holding vaccine doses fixed, we find the characteristic V and W shapes are shifted to the left. The number of vaccine doses needed to reach the critical herd immunity threshold increases as the basic reproductive number increases. Unequal approaches become more favorable as the level of underlying immunity in population 1 increases, because fewer doses are required for population 1 to reach their herd immunity threshold. Thus, even for very high *R*_0_ values, the optimal strategy, minimizing the cumulative number of cases across both populations, prioritizes allocating doses to the population that is closest to reaching its critical herd immunity threshold.

### 5.5 Impact of delayed vaccine roll-out in a homogeneous population

**Figure 4:**
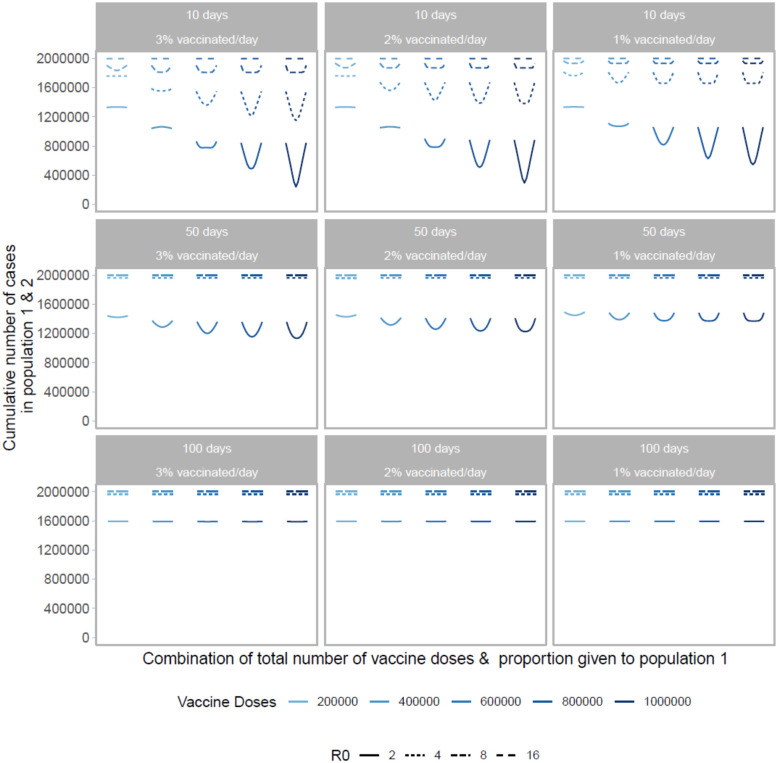
Performance of different allocation strategies of a limited SARS-CoV-2 vaccine stockpile across two homogeneous populations of equal size (one million individuals) with no underlying immunity, with vaccines rolled out at different speeds and different times after the start of the epidemic. Each color represents a different number of total vaccine doses. Each line represents a different basic reproductive number. We vary both the timing and speed of roll-out between 10, 50 or 100 days after the start of the epidemic with 1, 2, or 3% of the population vaccinated per day. Each column represents a given roll-out speed while each row represents a different timing.

Next, we examine the impact of vaccine roll-out over the course of the epidemic. We find both the timing and speed of roll-out play an important role in minimizing the final size of the epidemic. As shown in Figure 4, the cumulative number of cases across both populations is minimized when vaccine roll-out occurs as soon as possible after the start of the epidemic. Further, the final size is minimized when roll-out speed is increased, vaccinating a larger proportion of the population each day.

For the early and efficient roll-out (beginning 10 days after the start of the epidemic, at a rate of 2 or 3% of the population/day), the vaccination performance profile across possible allocations looks similar to that of the prophylactic vaccine deployment strategy shown in Figure 1. However, for a slower or more delayed roll-out we see highly unequal approaches perform poorly across almost all doses and more equal approaches result in the smallest final size. This is because a larger fraction of the population is naturally infected, minimizing the gains from concentrating vaccine doses in one population.

As we incorporate differences in transmissibility, we find timing and speed to be of greater importance. Even with a vaccine roll-out 50 days after the start of the epidemic, there are no differences in final size across all allocation strategies, within a given *R*_0_ level, as the epidemic has ended in the population before vaccines are introduced. For higher reproductive numbers, faster, earlier roll-outs are needed for vaccination to have an impact on the total number of infections.

### 5.6 Impact of heterogeneous population structure

Looking within a population, many studies have shown optimal strategies favor prioritizing older individuals (e.g., those aged 60 or over) when the goal is minimizing mortality. If the goal instead is minimizing final size, targeting adults 20–49 with an effective transmission-blocking vaccine minimizes cumulative incidence.^8,9^ Here we model the impact of heterogeneous population structure to examine the impact of strategies across populations. These simulations consider populations with heterogeneous transmission or with heterogeneous risk of death.

**Figure 5:**
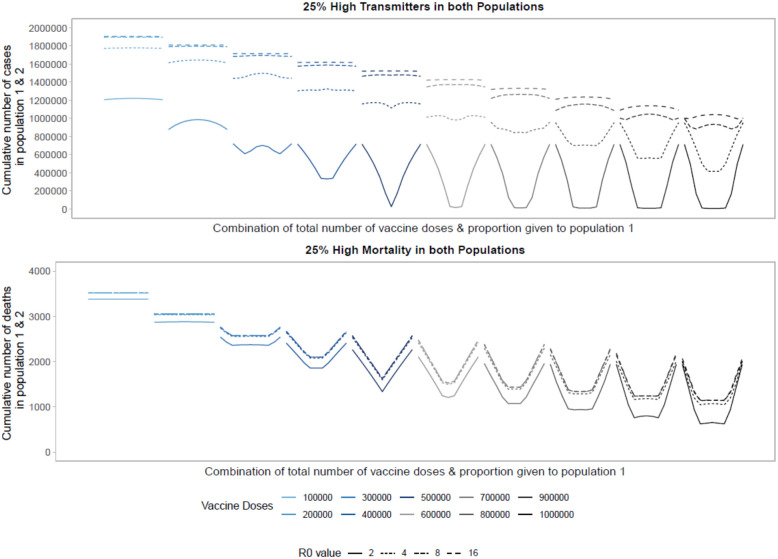
Performance of different allocation strategies of a limited SARS-CoV-2 vaccine stockpile across two heterogeneous populations of equal size (one million individuals) with no underlying immunity and prophylactic vaccination. Each color represents a different number of total vaccine doses. Each line represents a different basic reproductive number. In both the high transmission scenario (top) and high mortality scenario (bottom), 25% of both populations are high risk.

Targeting high transmission or high mortality groups first within a population shifts the optimal allocation across the two populations towards pro-rata allocation (Figure 5, S4, S5). In Figure 5 we first model the impact of prophylactic vaccination in a heterogeneous population structure with 25% of each population at either high risk of transmission (top) or death (bottom). In the high-transmission scenario, the behavior looks similar to that in Figure 1 for a low number of doses, representing the trade-off between vaccinating the high-transmitters in both populations. Once there are enough doses available to vaccinate enough high-transmitters to reduce transmission dramatically, the optimal strategy favors more pro-rata allocations across the two populations as high-transmitters are driving the bulk of transmission. This shift to more equal allocations occurs at a lower number of vaccine doses compared to Figure 1. In the high-mortality scenario, we see the optimal allocation rapidly shift to pro-rata strategies, starting at a very low number of vaccine doses. Interestingly, the sequence of profiles from Figure 1 is repeated twice. First, for a low number of vaccine doses there is a trade-off between vaccinating the high-mortality individuals in both populations. Then for higher vaccine counts the trade-off is repeated, this time between all individuals of both populations. While this trade-off exists, pro-rata allocation is heavily favored across almost all levels of available vaccine doses.

Looking across different levels of *R*_0_, we find similar trends. Vaccinating higher transmission or mortality groups first results in more equal allocation strategies across populations. For higher *R*_0_ values (i.e., 8 or 16) pro-rata allocation performs comparably to highly unequal strategies. Increasing the proportion of high-risk individuals to 50% of the population (Figure S3) we find similar trends for *R*_0_ values of 2 and 4. For higher *R*_0_ values, unequal approaches perform optimally as a larger fraction of the population is driving transmission, so effectively targeting this group in either population minimizes the cumulative number or deaths or cases across the two populations.

Next, we considered the impact of continuous roll-out for both the high transmission and high mortality scenarios. We find that across both high-risk scenarios and all vaccine roll-out times and speeds, unequal allocation is highly sub-optimal (Figure S4–S7). Similarly to Figure 4, we vary the start date of vaccination roll-out (1, 10, 30, 50, or 100 days), the daily vaccination rate (1, 2 or 3% per day), and the proportion of the population at high risk (25 or 50%). We find that both the speed and timing of vaccine roll-out are important factors in minimizing the cumulative number of cases or deaths across the two populations and see the greatest reduction in cumulative deaths and final size with the earliest and fastest roll-out. Specifically, for vaccine stockpiles larger than 500,000 doses, the achievable impact of vaccination is more dependent on the timing (solid vs. dashed curves) and speed (different panels) of vaccine roll-out rather than on the total number of doses available.

## 5.7 Sensitivity Analyses

We assessed the robustness of our results by varying the characteristics of the vaccine and connection between populations to be more representative of the current pandemic. As expected, the leaky and all-or-nothing vaccine have the same critical vaccination threshold, though the cumulative number of cases in the leaky vaccine scenario is equal to or larger than the all-or-nothing scenario^34^ (Figure S1).

In the previous situations we have only considered the scenario of non-interacting populations. As we relax this strict assumption, we find that as the amount of interaction between the two populations increases, equal strategies are most favorable (Figure S2). When the force of infection in each population depends on epidemic dynamics in both populations, accounting for interaction drastically changes the optimal allocation profiles and favors equal allocation between populations, as seen in previous work.^11,18^ Even for low values of the interaction parameter *i*, equal allocation rapidly becomes optimal. Indeed, for *i* values higher than 0.01 — which corresponds to one out of every hundred infected individuals contributing to infection in the other population — equal allocation between the two population always performs best. As *i* further increases, unequal strategies progressively approach the optimal (equal) allocation as indicated by the flattening of the curves. For *i* equal to 0.5, when the two populations concretely behave like one large population, all allocation strategies perform almost identically. Compared to the non-interacting case, allowing for interaction between the two populations leads to a higher cumulative number of infections for all possible vaccine allocation strategies, and the “W”-shaped allocation curve no longer appears.

As we increase the basic reproductive number, we find that the optimal strategy quickly favors more equal allocation decisions. In addition, interaction between the two populations becomes less important for very high values of R_0_, as the allocation profiles look similar across all interaction parameters for R_0_ values of 8 and 16.

## 6 Discussion

As countries continue to roll-out vaccines, challenging allocation decisions will need to be made due to resource constraints. Previous studies11 have shown simple scenarios favor unequal allocation. We recreated those findings, and further extend vaccine allocation theory, and apply it to the current COVID-19 pandemic.

In the simple case of two non-interacting populations of identical size we show that for very high quantities of vaccine, relative to population size, equal allocation strategies are optimal. For very few doses, all strategies provide comparable results. This supports the European Commission’s decision to allocate vaccine doses proportional to population size among the 27 European Nations.^35^ In this simplest model, until there is enough vaccine for both populations to approach their critical herd immunity threshold, optimal strategies favor a highly unequal approach, allocating doses to either population 1 or 2 until the population has reached its threshold. If the populations vary in size, allocation decisions vary, and as the number of vaccines increases, focus switches from the smaller population to the larger one, as supported by Keeling and Shattock.^11^

We consider more realistic scenarios that better mirror the current COVID-19 pandemic including underlying immunity, population interaction, continuous vaccine roll-out, heterogeneous population structure, and differences in underlying disease transmissibility (either because of biological or social factors). While many of these parameters are either unknown or changing throughout the course of the epidemic, we find that, across a range of scenarios, optimal allocation decisions often favor equal allocation across populations. Since these strategies are often optimal or nearly optimal across a range of parameters, while unequal allocations are only generally optimal for narrow parameter ranges, more pro-rata strategies might be the best option under uncertainty in an ongoing epidemic. Parameter values from COVID-19 illustrate this phenomenon; however, these results contribute more generally to the existing vaccine allocation theory for any epidemic emerging in multiple populations when key epidemic variables remain unknown.

For scenarios considering heterogeneous population risk, we find that first targeting high risk individuals, either high-transmitters or those at higher risk of death after infection, results in more equal allocations between populations being optimal. Targeting high-risk individuals first, then shifting priority to lower-risk individuals is supported by previous modeling work, looking at SARS-CoV-2 vaccine allocations within a single population,^8–10,36,37^ and is in concordance with the ongoing COVAX strategy, targeting early doses to high-risk individuals, and the USA’s implement which vaccinated health care workers and elderly individuals first.^38,39^

Our modeling analyses are subject to many simplifying assumptions on population dynamics and vaccine characteristics that may not be applicable to the current pandemic. We consider a vaccine that prevents both SARS-CoV-2 disease and infection, thus providing indirect protection to a fraction of the population. Vaccines appear to be able to reduce infectiousness, but this effect still needs to be precisely assessed. We do not model vaccine refusal and assume that all individuals given doses accept them. Recent studies^40^ show vaccine hesitancy as a threat to successful COVID-19 response. Next, we do not consider delays between doses, but model the epidemic from a final dose which confers 95% efficacy. Due to vaccine shortages, the delay between the first and second dose could impact our findings as individuals may be able to get infected in the interim. Further, we only model one available vaccine. The current SARS-CoV-2 vaccine landscape is complex, and multiple vaccine candidates are rapidly undergoing testing. Considerations for optimal allocation in this context are more complicated, especially if the vaccines have different immunogenicity profiles, and the quantity of doses and timing of roll-out varies across candidates. Further, we only consider one strain of SARS-CoV-2, not taking into account variants with varying transmissibility and mortality. Finally, we do not consider the impact of non-pharmaceutical interventions (NPIs) in conjunction with vaccination. Furthermore, we only model allocation strategies within two symmetric populations. It is likely that policy makers will face allocation decisions across multiple countries, or across multiple regions within a country. While our analyses do not extend to more than two locations, general principles should remain the same, as illustrated elsewhere for three populations.^11,18^

Future modeling work on SARS-CoV-2 vaccination strategies is needed, as multiple vaccine candidates continue to be rolled out. These studies should also consider the effects of vaccines on reducing hospitalizations and preserving hospital capacity, which may have indirect benefits for mortality rates for COVID-19 and other diseases beyond the direct prevention of infection in high-risk populations. In addition, other work should also consider populations with varying epidemic dynamics, and distribution capacity. Indeed, it has been argued that populations at higher immediate risk of SARS-CoV-2 spread and populations where vaccine roll-out is most efficient should be prioritized for SARS-CoV-2 vaccine allocation.^41^

With vaccine supplies still severely constrained, rapid allocation decisions will need to be made. Due to the magnitude of SARS-CoV-2 spread and impact globally, further political and economic constraints will likely play a large role in allocation decisions. Mathematical modelling can provide insight into optimal allocation strategies that maximize the benefit from each dose. Conclusions from such models should be balanced with ethical considerations on the fairness of allocation that also minimize disparities in access. We show key principles that should be considered in the design of realistic and implementable allocation strategies.

## Supplementary Material

Supplement 1
