# Supplementary material for: Comparative performance of between-population allocation strategies for SARS-CoV-2 vaccines
Source: medRxiv. 2021 Jun 21:2021.06.18.21259137. Preprint. [Version 1] doi: 10.1101/2021.06.18.21259137 (PMC8246345; doi:10.1101/2021.06.18.21259137)
Supplement: Supplementary file 1 [file media-1.pdf]

Supporting Information
Comparative performance of between-population allocation strategies for SARS-CoV-2 vaccines

Keya Joshi[1,*,a], Eva Rumpler[1,*,a], Lee Kennedy-Shaffer[a,b], Rafia Bosan[a] and Marc Lipsitch[a]

[a]Center for Communicable Disease Dynamics, Department of Epidemiology, Harvard TH Chan School of Public Health, 02115 Boston, Massachusetts
[b]Department of Mathematics & Statistics, Vassar College, 12604 Poughkeepsie, New York
[1]Contributed equally to this work.
[*]Corresponding authors.

A Supporting Information

A.1 Supplementary Figures

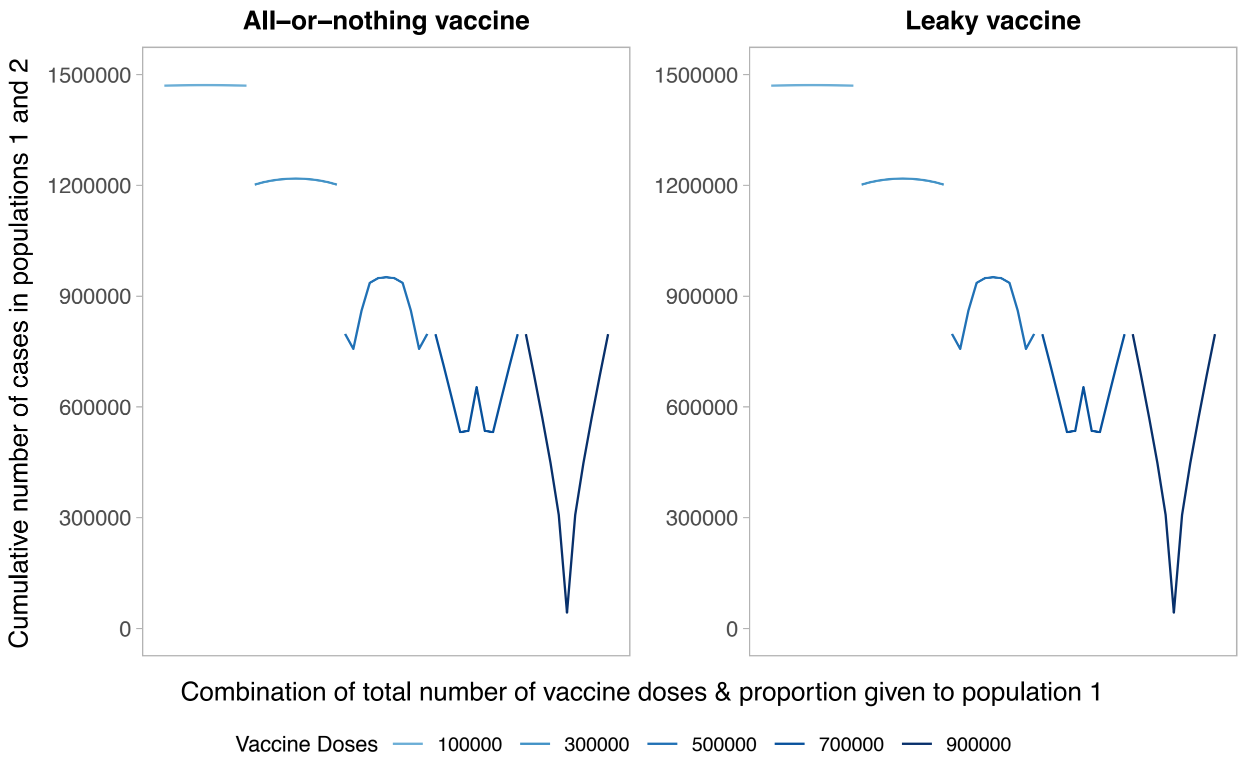

Figure S1: Performance of different allocation strategies of a limited SARS-CoV-2 vaccine stockpile across two homogeneous populations of equal size (one million individuals) with no underlying immunity and prophylactic vaccination. An all or nothing vaccine (left) is compared to a leaky vaccine (right). The panel on the left is equivalent to Figure 1.

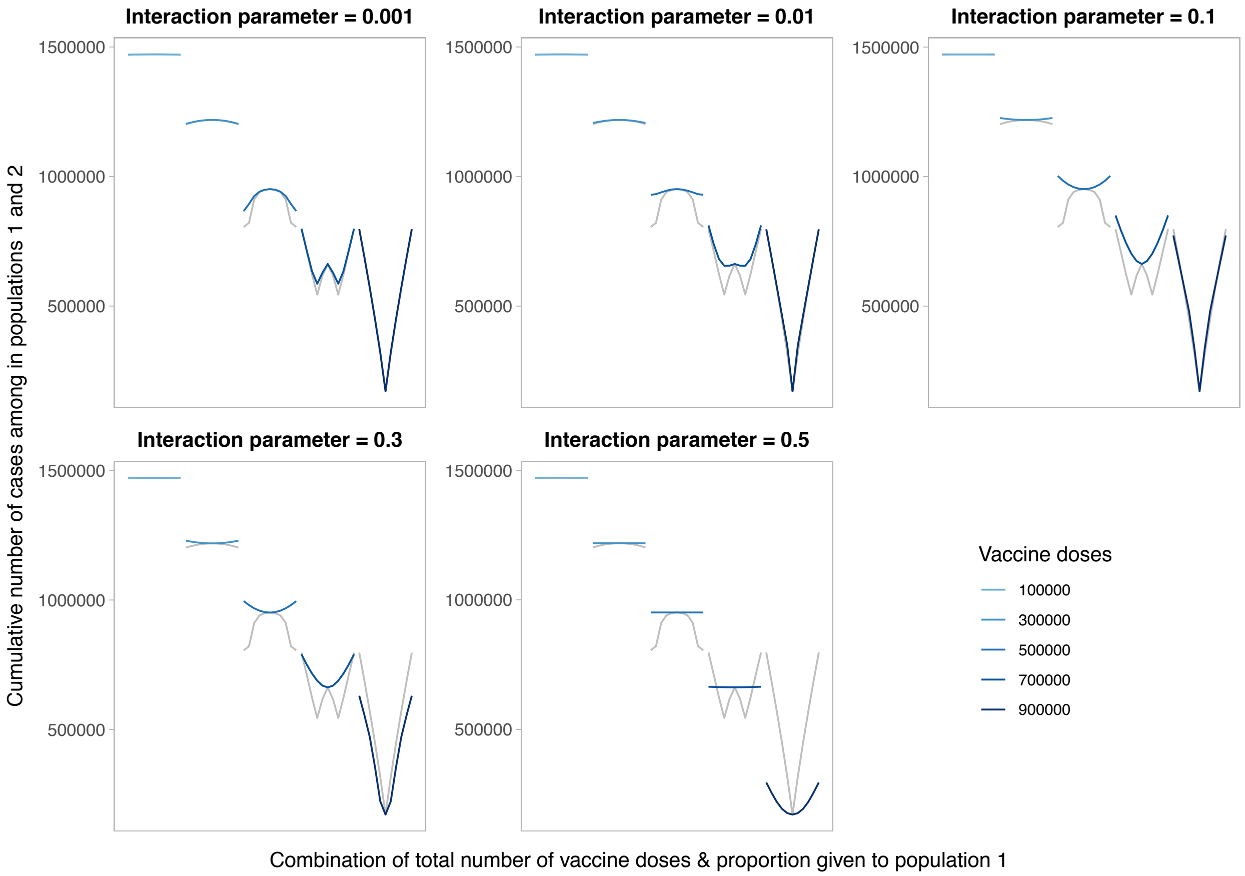

Figure S2: Performance of different allocation strategies of a limited SARS-CoV-2 vaccine stockpiles across two homogeneous populations of equal size (one million individuals) with no underlying immunity, with vaccines rolled out starting 10 days after the start of the epidemic with 2% of the population vaccinated each day. The populations are allowed to interact to a varying degree ($i = 0$ corresponds no interaction and $i = 0.5$ corresponds to complete interaction or perfect mixing between the two populations). The grey lines in each panel are equivalent to the solid lines in the middle panel of Figure 4 and represent a scenario without interaction between the two populations.

A.2 Model and Parameters

A.2.1 SEIR model equations for two non-interacting populations

Population 1

$$\frac{dS_1}{dt} = -\beta S_1 I_1$$

$$\frac{dE_1}{dt} = -\beta S_1 I_1 - \sigma E_1$$

$$\frac{dI_1}{dt} = \sigma E_1 - \nu I_1$$

$$\frac{dR_1}{dt} = \nu I_1$$

Population 2

$$\frac{dS_2}{dt} = -\beta S_2 I_2$$

$$\frac{dE_2}{dt} = -\beta S_2 I_2 - \sigma E_2$$

$$\frac{dI_2}{dt} = \sigma E_2 - \nu I_2$$

$$\frac{dR_2}{dt} = \nu I_2$$

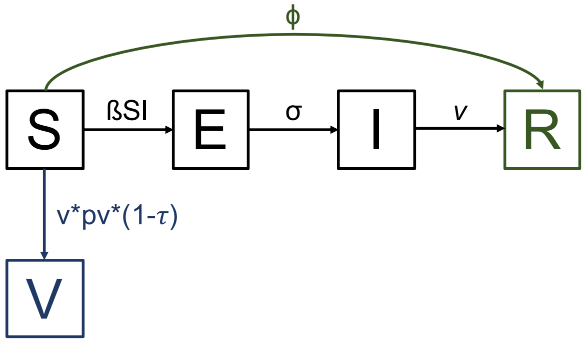

Figure S3: SEIR model incorporating underlying immunity (in green) and continuous roll-out of vaccination (in blue)

A.2.2 Parameters

Parameter	Definition	Value
σ^{-1}	Latent period	3 days
ν^{-1}	Infectious period	5 days
R_0	Basic reproduction number	2
τ	Vaccine efficacy	0.9
v	Number of vaccine doses available per population	[0,1,000,000]
pv_1	Proportion of the total vaccines given to population 1	[0,1]
ϕ_i	Proportion of the population immune in population 1	0, 0.1, 0.2, 0.4
Rollout time	Vaccine roll-out time	1, 10, 30, 50, 100 days
Rollout speed	Vaccine roll-out speed	1, 2, 3% per day
ph_i	Proportion of high risk individuals in population i	0.25
i	Proportion of interaction in population 1 and 2	[0,0.5]

A.2.3 SEIR model equations for two interacting populations

We extend the equations from the original SEIR model to allow for interaction between population 1 and population 2.

Population 1

$$\frac{dS_1}{dt} = -\beta S_1[(1-i)I_1 + iI_2]$$

$$\frac{dE_1}{dt} = \beta S_1[(1-i)I_1 + iI_2] - \sigma E_1$$

$$\frac{dI_1}{dt} = \sigma E_1 - \nu I_1$$

$$\frac{dR_1}{dt} = \nu I_1$$

Population 2

$$\frac{dS_2}{dt} = -\beta S_2[(1-i)I_2 + iI_1]$$

$$\frac{dE_2}{dt} = \beta S_2[(1-i)I_2 + iI_1] - \sigma E_2$$

$$\frac{dI_2}{dt} = \sigma E_2 - \nu I_2$$

$$\frac{dR_2}{dt} = \nu I_2$$

A.2.4 Global R0 calculation for heterogeneous populations

To identify the global basic reproduction number for the population in simulations with multiple types of individuals in the population, we use the next generation matrix for the SEIR model [1], with two compartments for each of the SEIR components, one for the high-risk individuals and one for low-risk individuals. Letting S_L^* and S_H^* denote the disease-free-equilibrium proportion of individuals in the low-risk and high-risk susceptible compartments, respectively, and letting β_{LL} be the force of transmission from one low-risk individual to another, β_{LH} from a high-risk individual to a low-risk individual, β_{HL} from a low-risk individual to a high-risk individual, and β_{HH} from one high-risk individual to another, we get the components of the next generation matrix:

$$F = \begin{pmatrix} 0 & 0 & \beta_{LL}S_L^* & \beta_{LH}S_L^* \\ 0 & 0 & \beta_{HL}S_H^* & \beta_{HH}S_H^* \\ 0 & 0 & 0 & 0 \\ 0 & 0 & 0 & 0 \end{pmatrix}$$

$$V = \begin{pmatrix} \sigma & 0 & 0 & 0 \\ 0 & \sigma & 0 & 0 \\ -\sigma & 0 & \nu & 0 \\ 0 & -\sigma & 0 & \nu \end{pmatrix}.$$

And thus:

$$V^{-1} = \begin{pmatrix} \sigma^{-1} & 0 & 0 & 0 \\ 0 & \sigma^{-1} & 0 & 0 \\ \nu^{-1} & 0 & \nu^{-1} & 0 \\ 0 & \nu^{-1} & 0 & \nu^{-1} \end{pmatrix}$$

$$FV^{-1} = \begin{pmatrix} \nu^{-1}\beta_{LL}S_L^* & \nu^{-1}\beta_{LH}S_L^* & \nu^{-1}\beta_{LL}S_L^* & \nu^{-1}\beta_{LH}S_L^* \\ \nu^{-1}\beta_{HL}S_H^* & \nu^{-1}\beta_{HH}S_H^* & \nu^{-1}\beta_{HL}S_H^* & \nu^{-1}\beta_{HH}S_H^* \\ 0 & 0 & 0 & 0 \\ 0 & 0 & 0 & 0 \end{pmatrix}$$

The spectral radius of FV^{-1} is then given by the spectral radius of the upper left 2×2 submatrix:

$$\begin{aligned}
\rho(FV^{-1}) &= \max\left\{|\lambda| : \begin{vmatrix} \nu^{-1}\beta_{LL}S_L^* - \lambda & \nu^{-1}\beta_{LH}S_L^* \\ \nu^{-1}\beta_{HL}S_H^* & \nu^{-1}\beta_{HH}S_H^* - \lambda \end{vmatrix} = 0\right\} \\
&= \max\left\{|\lambda| : (\beta_{LL}S_L^* - \lambda\nu)(\beta_{HH}S_H^* - \lambda\nu) - \beta_{LH}S_L^*\beta_{HL}S_H^* = 0\right\} \\
&= \max\left\{|\lambda| : (\nu\lambda)^2 - (\beta_{LL}S_L^* + \beta_{HH}S_H^*)(\nu\lambda) + \beta_{LL}S_L^*\beta_{HH}S_H^* - \beta_{LH}S_L^*\beta_{HL}S_H^* = 0\right\} \\
&= \max\left\{|\lambda| : \nu\lambda = \frac{\beta_{LL}S_L^* + \beta_{HH}S_H^*}{2} \right. \\
&\qquad \left. \pm \frac{\sqrt{(\beta_{LL}S_L^* + \beta_{HH}S_H^*)^2 - 4(\beta_{LL}S_L^*\beta_{HH}S_H^* - \beta_{LH}S_L^*\beta_{HL}S_H^*)}}{2}\right\} \\
&= \frac{\beta_{LL}S_L^* + \beta_{HH}S_H^* + \sqrt{(\beta_{LL}S_L^* - \beta_{HH}S_H^*)^2 + 4\beta_{LH}S_L^*\beta_{HL}S_H^*}}{2\nu}.
\end{aligned}$$

$$= \frac{R_{LL} + R_{HH} + \sqrt{(R_{LL} - R_{HH})^2 + 4R_{LH}R_{HL}}}{2}$$

This value is the global R_0.

R_{HL} and R_{LH} represent the number of secondary infections in high-transmitters generated by an infected low-transmitter and the number of secondary infections in a low-transmitter generated by an infected high-transmitter, respectively. Values on the diagonal represent R_{HH} and R_{LL} which are the number of secondary infections in high-transmitter members generated by an infected high-transmitter and the number of secondary infections in a low-transmitter generated by an infected low-transmitter.

A.2.5 Vaccine Allocation decision rules

Below we describe the decision rules when allocating a limited number of vaccines to two populations with heterogeneous risk structure.

- 1. Assign doses to either population 1 or population 2. This is determined by the pv parameter.
- 2. If the number of vaccine doses is greater than the population size ($v \times pv > N_1$) everyone in population 1 is vaccinated, and the leftover doses are assigned to the second population.
- 3. Once doses are assigned to each population, within each population we first assign all doses to the high-risk individuals and give any doses left to the low-risk individuals. This is accomplished by checking whether the number of doses are sufficient to cover all of the high-risk individuals in that population (i.e., $N_1 \times ph_1 < v \times pv_1$). If not, we assign all of the doses to high-risk individuals and none to low-risk individuals. If the number of doses are sufficient to cover all high-risk individuals, all of those individuals are vaccinated and the remaining doses are assigned to low-risk individuals.

A.3 Literature

A.3.1 Optimal allocation across populations papers

1st tab : https://docs.google.com/spreadsheets/d/1NsWWBcztpGG4IpU2U65cUUMryNW9IoKQnoWyd6zig50/edit?usp=sharing

A.3.2 COVID-19 modeling papers

2nd tab : https://docs.google.com/spreadsheets/d/1NsWWBcztpGG4IpU2U65cUUMryNW9IoKQnoWyd6zig50/edit#gid=39102622

A.3.3 Details on optimal allocation threshold

Duijzer et al. (2018) [2] identify a number of features of the direct and indirect effect of vaccination that determine the optimal threshold to which to vaccinate populations. The authors seek to minimize the final size of the epidemic or, equivalently, maximize the total number of people who escape infection. When the number of vaccine doses available is less than the herd immunity threshold, this is also equivalent to maximizing the number of susceptible individuals remaining at the end of the epidemic, i.e., the number of unvaccinated individuals who escape infection, denoted the "herd effect." They determine that the herd effect, as a function of the vaccination fraction f within a population, denoted $G(f)$, has a predictable structure: it is increasing and convex for a low value of f, until $\bar{f}$. From $\bar{f}$ to f^*, it is increasing and concave. Above f^*, G is a decreasing function. f^* is equivalent to the herd immunity threshold, equal to $1 - 1/R0$ in a fully susceptible population. Vaccinating beyond f^* decreases the herd effect, as individuals who would be (somewhat) protected through herd immunity are instead vaccinated and protected directly instead. This convex-concave structure occurs because, for low values of f, the epidemic peak is delayed in addition to being smaller in magnitude. Whereas for values of f closer to f^*, the epidemic peak is advanced and smaller in magnitude. As f increases to f^*, this earlier peak continues to advance, leading to a decline in the increase in the herd effect and a concave G function. The authors identify another quantity: the dose-optimal vaccination fraction, $\tilde{f}$, where $\bar{f} \leq \tilde{f} \leq f^*$. The dose-optimal vaccination fraction maximizes the increase in herd effect per dose of vaccine. They find that for multiple non-interacting populations, the optimal allocation is to vaccinate as many populations as possible to the level $\tilde{f}$, and not vaccinate any other populations (except for perhaps one with any extra doses).

However, Duijzer et al. (2018) [2] prove that these features do not hold when there are no active infections (e.g., prior to the outbreak). In this case, $\bar{f} = \tilde{f} = f^*$. That is, G is an increasing and convex function prior to the herd immunity threshold and a decreasing and concave function after the herd immunity threshold. In this case, the results of Duijzer et al. align with those of Keeling and Shattock (2012) [3]: the optimal allocation scheme is to vaccinate as many populations as possible up to the herd immunity threshold. The difference from the previously-described situation is that the peak is always delayed by increasing the vaccinations in a population with no active infections. Since pre-outbreak vaccination leads to a decreased transmission rate for all cases in the population, the reduced number needed to reach the pandemic peak is always outweighed by the increased time required to infect those individuals. Duijzer et al. (2016) [4] previously demonstrated that, in this case, maximizing the herd effect is achieved when $\text{Rt} = 1$, i.e. at the herd immunity threshold, so this comports with that finding as well.

References

[1] Pauline van den Driessche. Reproduction numbers of infectious disease models. *Infectious Disease Modelling*, 2(3):288–303, August 2017.

[2] Lotty E. Duijzer, Willem L. van Jaarsveld, Jacco Wallinga, and Rommert Dekker. Dose-Optimal Vaccine Allocation over Multiple Populations. *Production and Operations Management*, 27(1):143–159, January 2018.

[3] Matt J. Keeling and Andrew Shattock. Optimal but unequitable prophylactic distribution of vaccine. *Epidemics*, 4(2):78–85, June 2012.

[4] Evelot Duijzer, Willem van Jaarsveld, Jacco Wallinga, and Rommert Dekker. The most efficient critical vaccination coverage and its equivalence with maximizing the herd effect. *Mathematical Biosciences*, 282:68–81, December 2016.
